# For us by us: Instituting mentorship models that credit minoritized medical faculty expertise and lived experience

**DOI:** 10.3389/fmed.2022.966193

**Published:** 2022-10-21

**Authors:** Eraka P. Bath, Kathleen Brown, Christina Harris, Alma Guerrero, Daniel Kozman, Charles C. Flippen, Isla Garraway, Karol Watson, Langston Holly, Sarah M. Godoy, Keith Norris, Gail Wyatt

**Affiliations:** ^1^David Geffen School of Medicine, University of California, Los Angeles, Los Angeles, CA, United States; ^2^Department of Psychiatry and Biobehavioral Sciences, Semel Institute for Neuroscience and Human Behavior, University of California, Los Angeles, Los Angeles, CA, United States; ^3^Department of Radiological Sciences, UCLA Health System, Los Angeles, CA, United States; ^4^Division of General Internal Medicine, University of California, Los Angeles, Los Angeles, CA, United States; ^5^Developmental-Behavioral Pediatrics, UCLA Mattel Children's Hospital, Los Angeles, CA, United States; ^6^Department of Medicine, UCLA Health System, Los Angeles, CA, United States; ^7^Division of General Internal Medicine and Health Services Research, UCLA Health System, Los Angeles, CA, United States; ^8^Department of Neurology, UCLA Health System, Los Angeles, CA, United States; ^9^Department of Urology, UCLA Health System, Los Angeles, CA, United States; ^10^School of Social Work, University of North Carolina at Chapel Hill, Chapel Hill, NC, United States

**Keywords:** mentoring, academic medicine, underrepresented in medicine (UIM), diversity equity and inclusion (DEI), antiracism

## Abstract

The woefully low proportion of scientists and clinicians underrepresented in medicine (UIM), including members of African-American/Black, Hispanic/Latinx, American Indian/Alaska Native or Native Hawaiian/Pacific Islander communities, is well characterized and documented. Diversity in medicine is not only just, but it improves quality and outcomes. Yet, diversity in academic medicine remains stagnant, despite national recognition and urgent calls to improve diversity, equity, and inclusion across health sciences. One strategy that has shown to improve diversity in many sectors is high quality mentoring. While many institutions have adopted mentoring programs, there remains a lack of mentorship that is equitable, individualized, and sets a clear timeline for academic milestones that will position UIM mentees at the optimal trajectory for promotion and retention. A barrier to assembling these programs is the small number of UIM among the senior faculty ranks who are able to serve in this role, given the disproportionate burden to serve on a multitude of academic committees, task forces, and workgroups to fulfill institutional mandates to diversify representation. These time-consuming services, documented in the literature as the “minority tax,” are generally uncompensated and unaccounted for in terms of consideration for promotion, leadership positions, and other measures of career advancement. The Justice, Equity, Diversity, and Inclusion Academic Mentors (JAM) Council represents a novel, culturally responsive, and anti-racist approach to achieve a more equitable and inclusive institutional environment. This approach strategically leverages the intergenerational wisdom and experience of senior UIM faculty *via* time-protected effort with the overall goals of improving rates of promotion, retention, and career satisfaction of early career UIM colleagues. This community case study describes the rationale, resources needed, processes, and proposed workflow required to launch the JAM Council, as well as the major roles and responsibilities for JAM mentors and mentees, which may be considered by academic medical centers focused on improving diversity among the faculty ranks.

## Introduction

The COVID-19 pandemic, the demand for racial and health justice in response to the brutal murders of George Floyd, Breonna Taylor, and many others, and the uprisings that emerged around the country signaled a shift in the collective consciousness around the naming of structural and institutional racism in various sectors, including health (1). Nationally, numerous jurisdictions cited racism as a public health crisis, including several of the most prominent organizations within organized medicine (2). Additionally, the National Academy of Medicine, the American Medical Association, and the National Institutes of Health, as well as multiple scientific journals publicly affirmed the support for Black Lives Matter and the need for racial reckoning, highlighting urgent calls for equity in opportunity and representation among all ranks including the highest within each institution (3, 4). These conversations echoed throughout academic medical centers around the country and prompted faculty members who are underrepresented in medicine (UIM) to build community around shared and lived experiences of isolation, and historic and ongoing aspects of internalized, interpersonal, and structural racism, while simultaneously rallying to create actionable steps to counter these visible and invisible practices of discrimination and denigration (5).

The low proportion of UIM faculty is well characterized and documented (6–8). As demands for racial justice in health science and medicine amplify, workforce diversity has been called out as an ongoing challenge and urgent necessity for achieving health equity. Diversity in medicine is not only just, but it improves health outcomes, perceived quality of care, productivity and innovation (9–12). In fact, the Association of American Medical Colleges (AAMC) has underscored that increasing diversity of the biomedical workforce is a key strategy to mitigate health inequity nationally (13). Unfortunately, despite national recognition and urgent calls to improve diversity, equity, and inclusion in health sciences, vast underrepresentation is evident across many racial/ethnic and gender groups (10, 11).

One strategy that has improved diversity in many sectors is quality mentoring (12–15). In most academic institutions, there is insufficient effective mentorship of early career UIM faculty with these faculty typically receiving less mentorship than their white colleagues (12–14). While many institutions offer mentoring programs of some type, few offer mentorship that is culturally responsive, equity-centered, individualized, and sets a clear timeline for academic milestones that will position the UIM mentee at the optimal trajectory for promotion and retention (16).

Mentoring is extensively documented as a key component to professional development, satisfaction with personal achievement and work environment, and successful promotion in academic medicine (12–14, 17, 18). Yet, consensus on best practice varies by institution, discipline, and perspectives from mentees and mentors (12, 13). Popular mentoring models include dyadic mentoring by an experienced investigator or team-based models where a mentoring team brings different expertise. The mentorship relationship often includes support related to scientific writing and grantsmanship, teaching, promotion, and tenure processes, maintaining work-life balance, time management, and managing and leading a team. Institutional components, including the support of key leaders and dedicated resources, are also critical for sustaining mentorship programs (14). There is well-established evidence that investing in faculty mentoring programs ultimately benefits the academic institutions at the organizational level through improved faculty satisfaction, retention, and institutional climate (12, 13, 19).

## Best practices in mentoring programs for UIM faculty

Although effective mentoring is critical for academic success, there are limited studies documenting the structure and effectiveness of mentoring programs for UIM faculty (12, 13). More recently, studies that include the implementation or evaluation of UIM mentoring programs that explicitly addresses common obstacles rooted in racist or biased practices and structures have been described (20–24). Research has found that mentorship programs for UIM faculty must assess for relevant elements that include mentors' potential for bias and discrimination, previous mentorship training, experience with intersectionality and isolation, and service on diversity committees, among other factors (20, 21). Yet, most UIM “specific” mentoring programs follow traditional content and structure of general mentoring programs with the only difference being a focus of implementation with UIM faculty.

Few programs are sufficiently adapted and tailored for the specific needs and perspectives of UIM faculty. For example, an adapted program may include pairing mentors and early career faculty who may or may not have similar social characteristics and personal experiences, but often lack a culturally-responsive, intersectional, and equity-centered approach (2). Mentoring programs for UIM faculty are also typically evaluated with general mentoring outcomes, such as career satisfaction and climate assessments (2) but fail to include salient measures related to inclusion and belonging. Effective evaluation tools for UIM mentorship programs should include assessment of belonging and connectedness, reduced clinical responsibilities when engaging in diversity, inclusion, justice, and equity focused service, and other factors that disproportionately burden and tax UIM faculty (24). In short, there is a critical need for a new genre of mentoring that explicitly addresses UIM-specific challenges with mentees and is supported by institutional leadership (13).

## Culturally responsive mentoring matters

While many institutions have adopted mentorship programs for early career faculty, there remains a persistent lack of formal mentorship programs created with an equitable lens for UIM faculty. In particular, there is a lack of mentorship programming that equips UIM faculty with the specific skills needed to navigate the culture of academic medicine that has historically centered and favored the professional organization and socialization of white supremacy values and ideology (25). This set of white supremacy values and ideologies that typically center on the heteronormativity of white, male faculty shapes the discriminatory and oppressive experiences of UIM faculty (26, 27) and other marginalized groups who are often few within their departments and situated in predominantly white institutions.

The numerous obstacles encountered by UIM and women faculty are well documented and include: (a) explicit and insidious racism; (b) microaggressions; (c) devaluation of scholarly contributions, merit, and skillset by colleagues and administrators; (d) the burden of representing all minoritized populations; and (e) the continuous and disproportionate requests to serve on committees, task forces, and workgroups to fulfill institutional mandates to diversify representation that are generally uncompensated, undervalued and unaccounted for, in terms of promotion consideration and career advancement (i.e., minority tax) (24, 25, 27–29). These experiences of both blatant and perceived discrimination, vocational strain, role overload, and adverse life events have also been shown to impact the physical and mental health of UIM faculty (27, 28). Available studies underscore how work-related stress and resulting health conditions may contribute to early morbidity and premature departures of UIM faculty from the academy, further highlighting the urgent need for robust institutional interventions to retain these faculty (19, 27). Additionally, UIM faculty are less likely to be promoted, have leadership positions, and be awarded National Institutes of Health R01 level grants compared to their white peers even after adjusting for multiple potential confounders (30, 31).

Specific mentoring practices and strategies for UIM and other marginalized groups are essential to help navigate these barriers to academic success and promotion. Mentoring programs should be tailored to the multitude of barriers UIM faculty face that non-UIM faculty are unaware of or may deny (25). The mentoring needs and demands for early career UIM faculty far exceed the limited supply of UIM mid-career and senior faculty, and this paucity of senior person-power exacerbates the gap in available mentorship opportunities and networks for those most at risk for attrition (19). Thus, it is important that institutional leadership intentionally create a culture of inclusive excellence by acknowledging the UIM specific challenges that require support and investment for effective mitigation. Therefore, it is critical for institutions to invest in mentoring infrastructure as reliance on grants to run such programs constitutes an unfunded mandate, typically shouldered by UIM mid-career and senior faculty that aggravates the minority tax (24).

## Context

At the David Geffen School of Medicine, the University of California, Los Angeles (UCLA) a newly formed collective of Black, Latinx, and Native American (BLNA) faculty engaged in a more explicit dialogue with executive leadership centered on the plight of retention and attrition of UIM faculty, who are often traversing spaces with racism, bias, microaggressions, and exclusion. Together, the collective recognized the need for both top-down and bottom-up antiracist strategies to address the invisible and opaque and the visible dimensions of structural racism in the academic setting. Several potential initiatives emerged from the BLNA faculty collective, including a mentorship program for early career UIM faculty that builds on promising practices.

### Rationale: For us by us

Institutionalization of an innovative mentoring program was identified as one of several strategies that could be leveraged to address the high attrition, low career satisfaction, and obstacles encountered in the promotion process for UIM faculty, particularly early career faculty. Specifically, a mentoring program that explicitly addresses racism, biased practices, and evaluation structures that adversely impact UIM faculty was identified as a key solution. Institutional investment and structural accountability with concrete measures and resources to address historical and ongoing oppression and discrimination is required to support an antiracist and equity-minded mentoring program.

To address common but harmful experiences for UIM faculty, strategies were envisioned by BLNA faculty to create an innovative approach to a mentoring program that would: (1) be driven and developed by UIM faculty and non-UIM faculty allies, using an antiracist and intersectional lens to provide the tools and strategies to navigate UIM-specific barriers to success in academia; (2) credit, value, and monetarize the lived experience and expertise of UIM mid-career and senior medical faculty; (3) hold academic centers structurally accountable in taking steps to address the historic and ongoing oppression and discrimination of UIM faculty; and (4) formalize and institutionalize the program into a critical component of an academic medical center through the *sustained* investment of monetary and non-monetary resources. We hypothesize that expanding the number of culturally sensitive mentors, improving their visibilities, and enhancing skill development through an institutional initiative will benefit career development and strengthen the onramps and pathways toward promotion and retention of underrepresented and/or traditionally marginalized faculty. The long-term goal is to observe a longitudinal decrease in attrition of underrepresented and/or traditionally marginalized faculty, thereby expanding their overall representation and success at the associate and full professor levels.

## Justice, Equity, Diversity, and Inclusion Academic Mentors (JAM) Council

### Key programmatic elements and purpose

The Justice, Equity, Diversity, and Inclusion Academic Mentors (JAM) Council at UCLA is a mentorship program designed to offer an intensive 1-year training program with extensive coaching in mentorship for UIM faculty in academic medicine. The JAM Council is anchored in building a sense of community, networking, and sponsorship opportunities for UIM faculty. The JAM Council targets mentees at the instructor and assistant professor levels who desire a sustainable and rewarding career in academic medicine and/or health leadership. In addition to offering traditional academic career development mentoring, the JAM Council program pairs early career UIM faculty with middle career and senior UIM and non-UIM allied faculty to curate a sense of belonging and connection by equipping them with the skills needed to explicitly navigate effects of racism, discrimination, and biased practices on their professional development and socialization within academic medicine. These include understanding and evaluating structural barriers that adversely impact UIM faculty.

**Figure 1:**
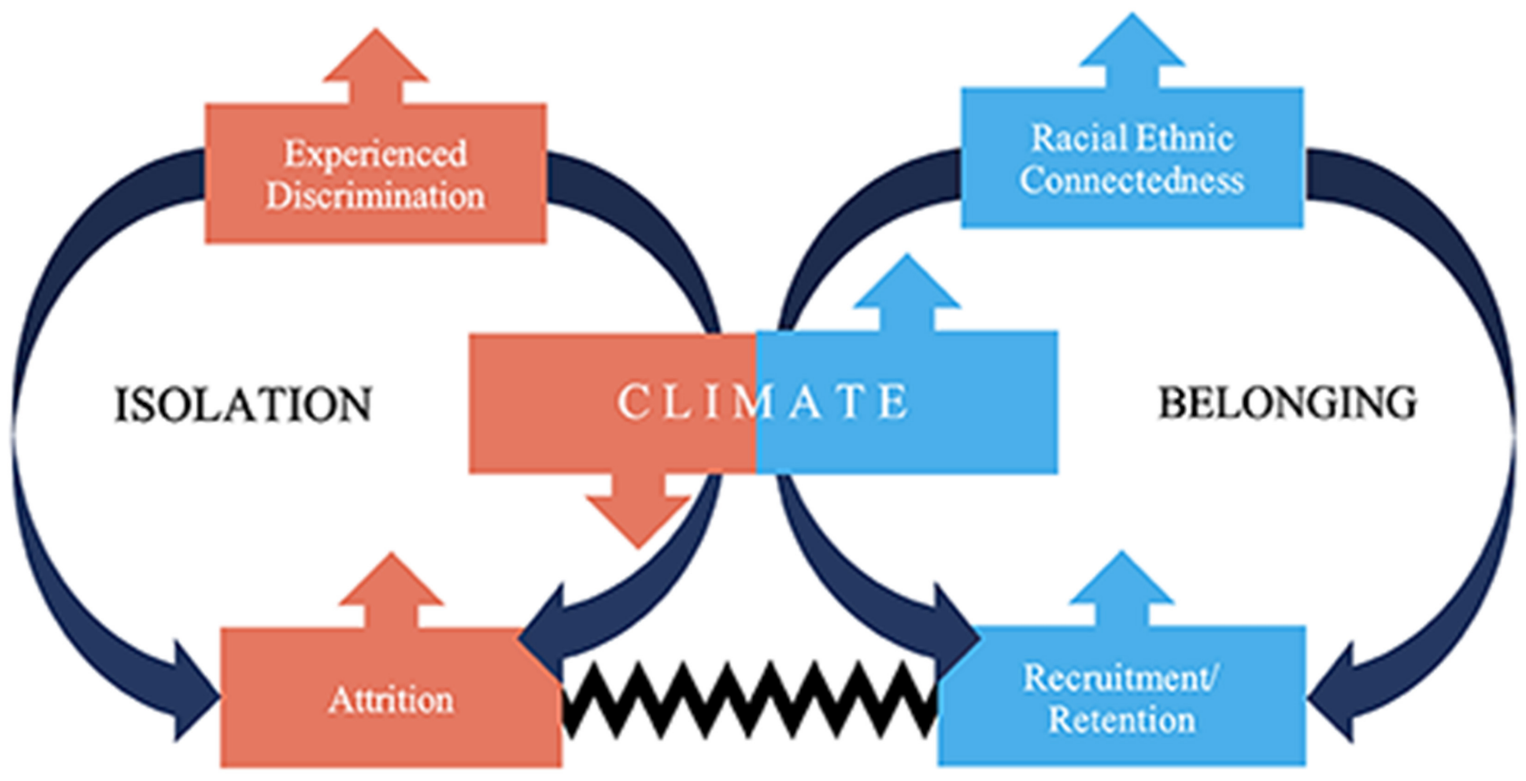
Model depicting how the JAM council can address negative workplace climates while promoting recruitment and retention, connectedness, and belonging.

Our conceptual model (see Figure 1) depicts how the JAM Council program aims to promote community building and belonging by connecting UIM faculty through a community of practice based on shared lived experience and intersectional identities. The program teaches UIM faculty how to navigate negative workplace climates, including experiences of discrimination and workplace isolation, the latter of which can result in a thwarted sense of belonging and lack of connectedness. Research has found that unequitable practices such as slower rates of promotion for UIM faculty, hostile workplace environments due to institutionalized racism and discrimination, and challenges finding effective mentorship are potent drivers of feelings of isolation, racial battle fatigue, and attrition (32–35) for faculty. Our goal is to increase retention and decrease attrition among UIM faculty, who are known to leave their institutions sooner at the assistant and associate professor level than their white colleagues, by specifically addressing belonging and promoting connectedness and inclusivity (19, 30). The white-UIM attrition gap is especially problematic given that UIM faculty are already severely underrepresented (36, 37) and face additional obstacles in academic structures, including workplace stress, distress of not fitting in, and imposter syndrome, which further exacerbate feeling unwanted in the workplace and social isolation (11, 26, 27). We posit that increasing creating affirming affinity spaces that enable connectedness based on shared racial and gendered minoritized statuses will help UIM faculty have a heightened sense of belonging and inclusion within their institutions and result in improved recruitment and retention of UIM faculty at various ranks.

### Structure

The JAM Council is composed of mentors within senior faculty positions from the various UCLA academic series and medical specialties. Mentors will serve 3-year terms as advisors and leaders of mentorship teams that will support early career UIM faculty appointed to departments within the UCLA David Geffen School of Medicine (DGSOM). JAM Council mentors include a group of faculty who represent a breadth of disciplines (e.g., surgical, non-surgical, biobehavioral, basic science) and academic series (e.g., research, clinician-educator). Mentors must fulfill several predetermined competencies to be considered for the position and will report directly to the Vice Dean of Faculty with *Ad Hoc* reporting to the Vice Dean of Justice, Equity, and Diversity Inclusion at the DGSOM.

Mentors must commit to supporting the scholarly productivity of prospective mentees and facilitate the development and advancement of their career goals. In addition to their assigned departmental mentors distinct from the JAM program, JAM council mentors will assist faculty mentees in preparation and development of a portfolio of research, teaching, service, and/or other scholarly activities to support promotion through the ranks, and to navigate an institutional environment still mired in racism and sexism, macro and microaggressions, conscious and unconscious biases. Mentor positions will be compensated by the Dean's Office within the medical school, rather than individual departments or other funding sources, with a stipend payable to the individual mentor at 10% of 2021 NIH cap (including benefits) depending on available effort, not too dissimilar from a NIH K-24 award mechanism for mentoring.

#### JAM Council mentors

There are several competencies and expectations for our JAM Council mentors (Table 1). Mentors must hold the rank of associate or full professor and must be supported by their Departmental leadership. The curriculum includes mentor training from National Research Mentoring Network, Center for the Improvement of Mentored Experiences in Research, as well as training in culturally responsive mentorship (20, 38). JAM Council mentors are responsible for: (1) providing personalized academic guidance and mentoring, (2) coordinating additional mentorship, sponsorship, and endorsement opportunities for UIM faculty from the time of their academic appointment, (3) assisting faculty mentees in preparation of a substantial portfolio of research, teaching, service, and other scholarly activities as needed for their series that will support promotion through the ranks, and (4) serving as a liaison with department promotion committee chairs to address any concerns about the dossier preparation.

**Table 1:** Competencies required for Justice, Equity, Diversity, and Inclusion Academic Mentors (JAM) Council Mentors.

M.D., PhD, or advanced doctoral degree required.
Professor or Associate Professor rank within their department.
Proven track record of and commitment to mentoring faculty from racial and ethnic groups underrepresented in medicine
Proven record in academic scholarship and leadership, as evidenced by publications, grants, speaking engagements, and positionality in organized medicine
Excellent interpersonal skills
Demonstrated ability to communicate effectively and work with all members of the academic and clinical community
Demonstrated understanding of racism (including structural racism), imposter syndrome, stereotype threat, microaggressions, intersectionality, and its power dynamics and the role of white supremacy ideology (versus individual identity) as the foundation for the creation and perpetuation of the racial caste system in America

The responsibilities span from general faculty development to focused preparation for early career faculty with expanded teaching roles, and activities that support those pursuing advanced training as education leaders, researchers, master clinicians, public health leaders, and scholars. This role is supplemental to the required departmental/divisional responsibilities for faculty success. JAM council mentors will participate in monthly meetings with their assigned mentee, monthly meetings with their mentoring team, and quarterly meetings with JAM Council. Expected time commitment for JAM mentors is ~4 h per week but could be more depending on the number of mentees.

The JAM Council advisory board will select mentor applicants based on their availability to devote the time necessary to participate in the program and their experience in mentoring those who identify as historically underrepresented in medicine and/or with historically marginalized identities. Mentors' applications will include answering a series of questions in the following areas: (i) self-reflection on interpersonal strengths and weaknesses as a potential mentor, (ii) history of mentoring and mentoring facilitation and how they might apply those skills to the JAM Council program, (iii) self-reflection on navigating identity and intersectionality and how/if they have impacted their academic journey thus far (whether as a student, trainee or faculty), and (iv) how they have supported the academic or career development for UIM mentees. The JAM Council advisory board reviewed all applications with a rubric that ranks their applicant responses across these four domains.

#### JAM Council mentees

All newly and recently appointed faculty at the instructor or assistant professor level are invited and eligible to apply as mentees to the program. Acceptance as a mentee into the JAM program is determined through an annual competitive selection process, in which ~20–25 candidates are chosen each year. Like the mentors, mentees are required to have the support of their departmental leadership. Mentees will participate in monthly seminars, monthly meetings with their assigned mentor and their mentoring cohort, and quarterly meetings with JAM Council advisory board. Expected time commitment for JAM mentees is estimated at ~4–6 h per month.

Similar to the process for selecting mentors, the JAM Council advisory board will select mentee applicants. Mentees will be evaluated based on their availability to devote the time necessary to participate in the program, overall career goals, and consideration of their experience in mentoring those who identify as historically underrepresented in medicine identities and/or with historical marginalized identities. Because one of the goals of the program is to expand workforce and build mentoring skills, consideration of mentee applicants' previous mentoring experience is included in application review. Mentee's applications included answering a series of questions in the following areas: (i) self-reflection on how they would benefit from the program, self-reflection on strengths and weakness and what they need to be successful in their current academic environment, (ii) scholarship and professional development goals, (iii) self-reflection on navigating identity and intersectionality and how/if they have impacted their academic journey thus far (whether as a student, trainee or faculty), and (iv) how they have been supported in the academic or career development as an underrepresented mentees. The JAM Council advisory board will review all applications with a rubric that ranks applicant responses across these four domains.

### Advisory board

The JAM Council advisory board is a six-member group of multidisciplinary faculty from the UCLA DGSOM Justice, Equity, Diversity, and Inclusion Oversight Committee that serve multiple critical roles. Their roles include: (a) the selection of mentees and mentors, (b) developing the core curriculum for the academic year, (c) meeting with mentees and mentors on a semi-annual basis for feedback about the program, (d) establishing metrics of success and deliverables for the program, and (e) interfacing with decanal leadership regarding resource requirements and progress of the program.

### Programmatic themes and curriculum

Thematically, the overarching content of the JAM Council focuses on mentoring early career UIM faculty to navigate invisible and visible acts of racism in academia, creating racial and ethnic connectedness, and establishing a safeguard and form of advocacy for non-UIM who are unaware of or deny the presence of barriers for the retention of UIM faculty. The JAM council and its multiple components were intentionally created around four programmatic themes derived from a series of meetings driven by BLNA faculty members and supported by decanal leadership. These themes are as follows: (a) it takes a village: creating a team of sponsors and champions, (b) level setting with transparent expectations: learning the rules of the game, (c) building bias resistance and resilience, and (e) fostering community, through team-building practices that promote connectedness and interdependence (Table 2).

**Table 2:** Key programmatic themes of the Justice, Equity, Diversity, and Inclusion Academic Mentors (JAM) program.

**It takes a village: creating a team of sponsors and champions**
One-on-one and small group new faculty orientation
Assembling/Coordinating a group of mentors across clinical, teaching, and research sectors to facilitate faculty development
Advisory to faculty for balancing teaching, research, and clinical roles
Help develop longitudinal mentoring between individuals at different stages in their academic career (e.g., Instructor, Assistant Professor)
Cultivate a mentoring team of near peer and senior mentors
Leveraging social capital to build mentee structured support network
**Level setting with transparent expectations: learning the rules of the game**
Detailed interrogation of expectations outlined in institutional promotion criteria
Rigorous dossier review and transparent level setting around areas in need of additional support
Mentor formal liaison with the departmental Committee on Academic Promotions
Strategies to navigate intersectional barriers in academic promotion
**Building bias resistance**
Help deconstruct for early career faculty the many dimensions through which white supremacy ideology operates so they can better navigate the structures and narratives in the academy that overtly or implicitly limit and marginalize faculty from racial and ethnic groups underrepresented in medicine.
Review common areas of “invisible racism” such as salary equity, clinical and teaching load, appropriate series pathway, leadership appointments, unpaid or underpaid adjunct activities as well as creating an individualized/tailored strategic plan to best navigate and/or counter any biased practices
**Fostering connectedness/interdependence**
Participate in longitudinal, intentionally designed community-building events that are both professional development related as well as enhancing social networks
Foster both formal and informal opportunities for mentee cohort to socialize and stay connected
Provide an environment where faculty's professional interests are fostered and valued for diverse contributions and potential desire to conduct health disparities or minority health focused research as well as to care for patients who often belong to minoritized groups and/or who are often uninsured or underinsured
Foster a safe, healthy, and supportive environment for faculty to maximize both personal and professional development and assist in achieving work-life balance.
Regular faculty development series in collaboration with the Office of Justice, Equity, Diversity and Inclusion.

The JAM Council is composed of a 16-session curriculum combined with individual, small team, and group mentoring delivered throughout the year (Table 3). The curriculum includes didactics, multimedia presentations, breakout sessions, group work, and role playing. Senior faculty mentors are part of a mentoring team with up to two to three mentees each. Early career mentees will be supported in a scholarly project that helps them develop and advance skills aligned with attaining their individual career goals. The JAM council curriculum addresses the following well-described threats to UIM faculty retention as well as key elements identified by the BLNA faculty group. Key curricular topics include but are not limited to: (a) the minority tax and service obligation—what it looks like and how to manage it; (b) promotion–important milestones and anticipating invisible criteria (e.g., the hidden curriculum) (39); (c) salary negotiations and personal wealth differential; (d) sponsorship; (e) intersectionality, isolation, code-switching, stereotype threat, and imposter syndrome; (f) gate blocking—anticipating biased, institutional factors at various levels of your career; and (g) negotiating equity in clinical spaces for UIM faculty. For example, UIM are more likely to care for financially marginalized patients (40), and also generate less income than their white counterparts (41). Although this fulfills institutional commitments to address health equity, this often results in lower compensation despite providing mission critical care that is more time-intensive.

**Table 3:** JAM council 16 session curriculum.

**Training Session**	**Topic**
Session 1	Establishing the Foundation: A Culturally Congruent Foundation for Mentoring and Navigating Intersectional Barriers
Session 2	Structural racism and challenges for disproportionately high number of faculty from groups underrepresented in medicine (UIM) in academia (e.g., first generation in medicine), racism, misogyny, finances (debt), stereotype threat, and minority tax
Session 3	Understanding how exceptionalism among UIM is used as a post-colonial like strategy to maintain white supremacy narratives of innate UIM inferiority but now with anointing a few exceptions
Session 4	The minority tax and service obligation—what does it look like and how do you manage
Session 5	Promotion—important milestones and anticipating the “invisible” criteria that may be applied to non-UIM faculty
Session 6	Financial Well-Being in Academia: Salary negotiations and personal wealth differential
Session 7	Sponsorship
Session 8	Intersectionality, isolation, and imposter syndrome
Session 9	Gate Blocking which includes anticipating biased institutional-level factors at various stages of your career
Session 10	Navigating clinical spaces for UIM—potential impact of treating more financially marginalized patients who generate less revenue leading to lower compensation despite providing clinical care that often requires more time—if the institution states this is part of its mission how to ensure equity in being valued and compensated for helping the institution reach its goals
Session 11	Physical Well-Being in Academia: Vocational strain & self-care
Session 12	Exploring and avoiding the minority tax
Session 13	Work-Life Balance in the Academy: Fact or Fiction? The additional “balls to juggle” for many UIM and faculty with other marginalized identities in trying to achieve balance
Session 14	Mentoring the next generation of clinician educators and basic scientists with an awareness of and dedication to achieve health equity

### Evaluation

Measurement tools specifically designed to evaluate formal mentorship programs in academic settings will be used. Because the goal of the JAM Council is to explicitly address the effects of racism, biased practices, and structures that adversely impact UIM faculty, in addition to traditional outcome measures such as career satisfaction, attrition and length to promotion, measurement of minority tax burden, belonging, connectedness, wellbeing, and impostor syndrome will be conducted. Evaluations will be conducted at multiple time points and include mixed methods of qualitative and quantitative measures with the goal of generating a holistic perspective to provide robust assessment of program processes, outcomes and lessons learned (20, 21).

## Discussion

In most institutions, there are few programs designed to increase diverse faculty in academic medicine with insufficient or incomplete mentorship available to early career UIM faculty (13, 15). While many institutions have adopted mentoring programs of some type, there remains a lack of mentorship for UIM faculty that is inclusive and equitable, culturally responsive, and anti-racist in explicitly addressing historic and ongoing aspects of racism (internalized, interpersonal, and structural). Also lacking are strategies on how to best cope with these same issues outside of the academic institution that further impact faculty wellness and productivity. Thus, the JAM Council was designed to leverage the experience and wisdom of senior UIM faculty to positively impact promotion, retention, and career satisfaction of early career UIM colleagues and to recognize and address the complex interaction of these factors.

In recognition of this critical need, the BLNA secured institutional support and commitment to the monetary and non-monetary resources necessary to conduct this activity. Institutional support has been repeatedly cited as a way to sustain mentorship programs, so they become embedded within academic health centers (13, 25). Institutional support is imperative to dismantling racism in medicine and academic by supporting programs and efforts that explicitly work against exclusionary practices that unfairly advantage white faculty by hiring, promoting, assigning higher value to their work, and enabling access to resources while disadvantaging UIM faculty (19, 25, 42, 43). Thus, by leveraging the power and privilege embedded in a predominately white institution to change the academic climate to be more supportive of advancement and productivity of UIM faculty the JAM Council fosters greater justice, equity, diversity, and inclusion (25, 44).

To ensure the success of the program, we have considered addressing structural and institutional threats that UIM faculty face over their academic careers. The first threat directly relates to the lack of racial and ethnic representation in academia. Given the consistently low number of UIM faculty in most academic settings, it is highly likely that their colleagues and administration will be of a differing race and/or ethnicity, and belong to a dominant group with conscious and unconscious negative biases toward the UIM faculty. This discordance has the potential to produce strained cross-cultural communications, due to differing lived experiences as well as professional and social frames of references. This can result in the UIM faculty member lacking the social connections necessary to build the supportive and collaborative environment needed to succeed in academia. Negative workplace environments and resultant social isolation can have a profound impact on mental wellness and ultimately academic productivity (45, 46).

The JAM Council was intentionally designed to counteract the threats posed by the lack of diversity and representation in academic medicine. The social isolation and lack of sense of belonging experienced by UIM faculty in academic medicine can have detrimental impact on their overall health, career progression, and success (27, 48–50). By ensuring that some mentors are also UIM faculty, we are attempting to bridge potential cross-cultural communication divides to facilitate trusted relationship rooted in the shared experience of membership in historically marginalized racial and ethnic groups in our racialized society. This racial concordance portends a lower likelihood of assumptions based on stereotypes, bias, or prejudice, thus allowing for a more genuine and trusted relationship (47). In fact, we posit that the connections formed by the JAM Council can serve as a form of bias resistance that can build bias resilience, both individually and as a collective. A unique aspect of the mentoring program will be its ability to promote racial and ethnic connectedness, and collectivism through a shared learning community.

It is important to highlight that although a goal of the JAM council is to build community and promote connections among UIM faculty mentors and early career/junior faculty scholars, the JAM Council program also includes non-UIM faculty mentors, who are equally pivotal to its successful implementation and sustainability. An overarching goal of the JAM Council program is to enhance workforce development and capacity in the area of inclusive and culturally mentoring practices, with an emphasis on groups who identify as UIM and/or have historically marginalized identities. The JAM Council program's curriculum intentionally embeds antiracism, and culturally responsive and informed mentoring techniques in its implementation (20). These include but are not limited to creating shared learning space where both mentor and mentees reflecting on their positionality along axes of power and privilege (20), and anchoring the program within historical context of understanding structural racism and bias in academy. Examples include using vignettes and videos to create reflective dialogue and role playing to address microaggressions.

The JAM Council may also catalyze research that advances health equity and potentially increase successful grant funding for UIM researchers. This may be especially salient given that prior research has shown that topic choice contributes to the significantly lower rate of NIH awards to African American/Black scientists compared to their white colleagues (31, 47). Further, funded UIM research scientists are more likely to conduct research that centers UIM communities. Their mentorship may lead to increases in funding for early career colleagues that share a research interest relevant to their community but may not understand the nuances of NIH and other study sections around funding priorities within the space of health equity research (13, 47).

### Limitations

Although we expect the JAM Council to significantly enhance justice, equity, diversity, and inclusion efforts within our institution, there are potential challenges and limitations with this nascent program. Due to limitations in resources and available mentors, a finite number of mentee positions are available. Presently, there is no other program available to early career faculty that are not selected for the mentorship program. Lastly, there are a relatively small number of senior UIM faculty available to participate as mentors. This potential concern is manifested in two distinct ways: (a) the program could add to the minority tax burden already carried by most of the senior UIM faculty; and (b) the need for a breadth of clinical disciplines and academic series among the senior mentors may be difficult to achieve with a relatively small number of mentors. These concerns are partially mitigated by decreasing the percentage effort required for the mentors, as well as including some non-UIM senior faculty to serve as senior mentors and add additional clinical discipline and academic series diversity.

## Conclusion

Enhancing retention of UIM faculty hinges upon improving positioning for leadership and advancement pathways, and this requires mentorship, sponsorship, and coaching that are institutionally supported and championed. Inadequate time and support must be addressed for UIM faculty to mitigate minority tax and other systemic barriers to provide and receive the critical guidance for producing the scholarship and innovation required for promotion (25, 49, 50). This requires true institutional commitment, from recognition of the problem to supplying the needed support and resources for programmatic success. This is not something that arises de novo within an institution and is unlikely to happen if championed by only a few faculty. The BLNA faculty collective sent a strong message to (a) identify the problem, (b) provide rationale for the solution, and (c) underscore likely outcomes of no action and action and were supported by executive and decanal leadership to operationalize this program. Fortunately, the JAM Council program has received significant support, resources, and executive sponsorship from the Dean's office who have included the JAM Council's program within their larger program of antiracism and addressing systemic racism across the medical school and health system. The JAM Council was co-created by the executive leadership in the Dean's office and has multiple touchpoints with the Deans of JEDI and the Deans for faculty, the latter of which are specifically attuned the requirements for advancement and promotion. The Dean's office has provided vital and necessary support which includes dedicated administrative staff, protected time for senior faculty to administer the program, space for holding in person meetings, IT support, public announcements, and resources (e.g., space, food, technology, and scheduling support) for hosting community building events. Broad dissemination by the Dean's office describing the program's significance and purpose has been an important part of the implementation strategy and buy-in has been communicated across the decanal and administrative structure. Updates on the JAM Council Program's progress will be embedded in standing executive meetings. This support is an essential element that reflect the institution's overall commitment to addressing structural racism and underscore their investment and buy-in to the process at the most senior ranks. It is important to note that the JAM Council is also launching within the medical school's larger Antiracism Roadmap, an overarching planned commitment to create structural accountability in antiracism in academic medicine. This includes but is not limited to updated guidelines to address bias and discrimination in the workplace and learning environment.

While it seems implicit, it can be challenging for majority groups to understand the isolation of marginalized and underrepresented groups in the same environment, let alone appreciate the need for community building. BLNA recognized that community is the cure for this isolation. Thus, there is a high need for community and affinity group spaces to be cultivated for UIM faculty within the academic medicine institutional environment. While affinity group spaces exist, they are not well recognized or structurally enfranchised. For instance, there may be research communities, including clinical research or global research, or education communities where people with shared needs come together. Doing the same for people of shared racial/ethnic identities is often labeled as reverse racism when in fact it is a response to implicit and explicit racism and discrimination that the majority are either unaware of or refuse to acknowledge. The implementation of the JAM Council will hopefully push the field of mentoring and anti-racism in medicine forward using an existing but unrecognized approach of cultivating microenvironments dedicated to faculty success. The JAM Council's evaluation will hopefully move such programs beyond perspective pieces by applying them and putting them into action.

## Data availability statement

The original contributions presented in the study are included in the article/supplementary materials, further inquiries can be directed to the corresponding author/s.

## Author contributions

EPB, CH, KB, AG, and KN contributed to conception and design of the article. EPB, CH, KB, AG, SMG, and KN wrote the first draft of the manuscript. All authors contributed to manuscript revision, read, and approved the submitted version.

## Funding

Funds for Open Access publication fees were received from UCLA DGSOM office of JEDI.

## Conflict of interest

The authors declare that the research was conducted in the absence of any commercial or financial relationships that could be construed as a potential conflict of interest.

## Publisher's note

All claims expressed in this article are solely those of the authors and do not necessarily represent those of their affiliated organizations, or those of the publisher, the editors and the reviewers. Any product that may be evaluated in this article, or claim that may be made by its manufacturer, is not guaranteed or endorsed by the publisher.
